# Impact of Obesity on Target Organ Damage in Patients with Metabolic Syndrome

**DOI:** 10.3390/diagnostics14141569

**Published:** 2024-07-19

**Authors:** Svetlana Kostić, Ivan Tasić, Nikola Stojanović, Jelena Rakočević, Marina Deljanin Ilić, Dragan Đorđević, Viktor Stoičkov, Isidora Tasić

**Affiliations:** 1 Institute for Therapy and Rehabilitation “Niška Banja”, 18205 Niška Banja, Serbia; kostic.ceki@gmail.com (S.K.); dr.ivan.tasic@gmail.com (I.T.); mdeljanin57@gmail.com (M.D.I.); ddj964@gmail.com (D.Đ.); viktorstoickov67@gmail.com (V.S.); 2 Faculty of Medicine, University of Niš, 18000 Niš, Serbiaitasic5@gmail.com (I.T.); 3 Institute for Histology and Embryology “Aleksandar Đ. Kostić”, Faculty of Medicine, University of Belgrade, 11000 Belgrade, Serbia

**Keywords:** metabolic syndrome, obesity, abdominal obesity, body mass index, waist circumference

## Abstract

Background: Metabolic syndrome (MetSy) is characterized by the presence of obesity, hypertension, altered glucose metabolism, and/or increased non-HDL cholesterol. This study aimed at elucidating the association between obesity with subclinical target organ damage and biochemical parameters included in MetSy pathogenesis. Methods: This study included 130 apparently healthy subjects. Plasma levels of oxidized-LDL-cholesterol (ox-LDL-Chol), nitric oxide (NO) metabolites, inducible NO synthase (iNOS), and plasminogen activator inhibitor-1 (PAI-1) were measured. Non-invasive assessment of liver disease included fatty liver index (FLI) and nonalcoholic fatty liver disease (NAFLD) fibrosis score. Carotid artery plaques were assessed by color Doppler imaging. Results: A total of 65 patients with MetSy were included in the MetSy group, while 65 without MetSy entered the control group. Ox-LDL-Chol levels were higher in the MetSy group compared to the control group, regardless of obesity. Levels of NO metabolites were similar in obese and non-obese patients with MetSy, but lower than in the control group. Obese patients with MetSy had higher iNOS values compared to non-obese ones, with similar PAI-1 levels. NAFLD was present in all obese patients with MetSy compared to 70% of non-obese subjects. Hypertension, higher values of waist-to-hip ratio, PAI-1, and remnant cholesterol were associated with NAFLD. Finding of asymptomatic carotid plaques was associated with patients’ age, hypertension, and higher waist-to-hip ratio. Conclusion: MetSy and obesity significantly alter the levels of NO metabolites, iNOS, ox-LDL-Chol, and PAI-1. High prevalence of NAFLD in obese patients with MetSy requires active screening and treatment of potential risk factors.

## 1. Introduction

Metabolic syndrome (MetSy) is defined as a set of cardiovascular risk factors that, when present, increase the risk of cardiac diseases, stroke, and diabetes mellitus [1]. The fundamental concepts linking MetSy into an important metabolic entity are abdominal obesity, hypertension, insulin resistance, and dyslipidemia. The latest definition of MetSy includes the presence of obesity, with at least two of the following criteria: hypertension, glucose metabolism disorder, and increased non-HDL cholesterol level [2]. Obesity represents an energy homeostasis disorder manifesting as excess body fat deposition. Body mass index (BMI) is the most widely used formula for the assessment of body fat amount, where values of 25–29.9 kg/m^2^ indicate overweight, and BMI ≥ 30 kg/m^2^ imply the presence of obesity. Although BMI is not a true measure of adiposity, this parameter is easily used in health screenings and epidemiological surveys [3].

BMI does not provide information regarding the fat tissue distribution, whether being visceral or femoral-gluteal. The presence of ectopic fat and/or visceral fat tissue is the key for the MetSy pathogenesis [2]. Therefore, waist circumference (WC) measurement is required to better estimate distribution of the body fat. WC represents a body size measurement that involves subcutaneous and visceral fat tissue surrounding certain visceral organs, skeletal muscles, and bones. Although WC strongly correlates with BMI, studies showed that WC may increase or exceed the predictive risk of diseases compared to the measurement of BMI alone [4].

Contradictory results on the relationship between BMI and WC have obliged us to include WC measurements in individuals with BMI between 25 and 34.9 kg/m^2^, as proposed by the current guidelines. Therefore, WC measurement may further improve the risk predictions, where high values (>88 cm in women, and >102 cm in men) significantly increase the risk of metabolic complications [5,6].

Obesity and MetSy strongly affect many organs, including heart, blood vessels, liver, and thyroid gland. Besides the well-known joint effect of obesity and MetSy on cardiovascular system and liver function, it has been shown that these two entities may increase the volume and the function of thyroid gland even in the eurthyroid population without clinically evident thyroid disease [7].

It is known that abdominal obesity and MetSy are strongly associated with inflammation and elevated values of numerous proinflammatory markers [8]. Oxidized LDL cholesterol (ox-LDL-Chol) has a central role in the development of atherosclerosis and can be regarded as a marker of lipoprotein-associated oxidative stress [9]. Ox-LDL-Chol is essential in different stages of atherosclerosis, including foam cell formation, leukocyte recruitment, and endothelial cell injury. On the other hand, there is a bidirectional relationship between ox-LDL-Chol and MetSy, with higher ox-LDL-Chol concentrations associated with higher risk of MetSy, and MetSy potentiating the oxidation of LDL [10], together leading to higher cardiovascular risk. Nitric oxide (NO) represents an important regulator of vascular tone and blood flow via dilatation of vascular smooth muscle cells [11]. In patients with MetSy, there is a reduced bioavailability of NO and its metabolites synthesized via constitutive enzyme endothelial nitric oxide synthase (eNOS) [12]. Ongoing inflammation during MetSy upregulates the expression of additional NO-synthesizing enzyme, inducible nitric oxide synthase (iNOS), which further promotes insulin resistance. Plasminogen activator inhibitor-1 (PAI-1) is an important attenuator of fibrinolysis, acting as an inhibitor of the plasminogen/plasmin system. However, it is well known that PAI-1 is overexpressed in patients with obesity, insulin resistance, and MetSy, and often it is considered one of the components of the metabolic syndrome [13].

Given the scarce data on the association between BMI, abdominal obesity, and target organ damage (such as blood vessels, heart, and liver), this study aimed at elucidating the association between the obesity with the risk of early subclinical target organ damage and specific biochemical parameters (ox-LDL-Chol, NO metabolites, iNOS, and PAI-1).

## 2. Materials and Methods

### 2.1. Subjects

**Figure 1:**
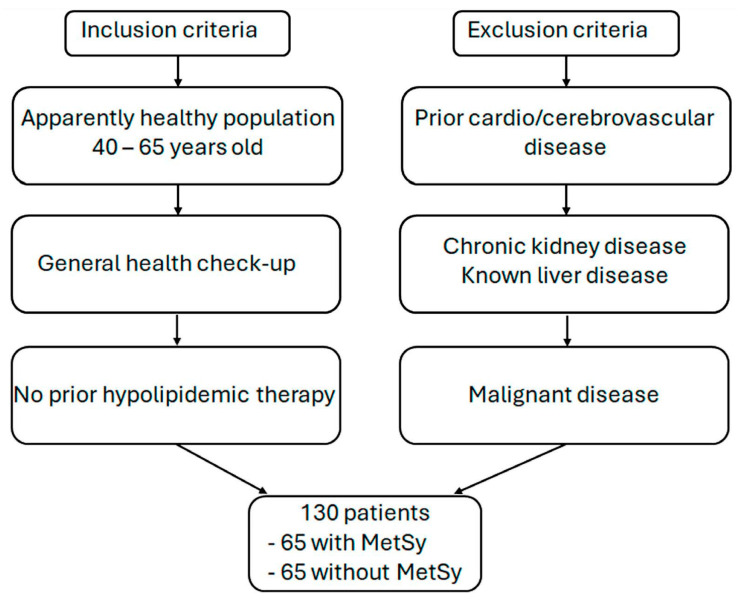
Inclusion and exclusion criteria for study cohort formation.

The study involved 130 apparently healthy subjects of both genders, aged 40–65 years, attending a general health check-up in the Institute for Treatment and Rehabilitation “Niška Banja”, Serbia, from February 2020 to May 2021. Their initial laboratory findings did not indicate the presence of any acute illness and acute cardiovascular disease. Exclusion criteria were as follows: (a) prior use of hypolipidemic therapy, (b) prior cardiovascular disease, (c) prior cerebrovascular disease, (d) chronic kidney disease, (e) known liver disease, and (f) malignant disease (Figure 1). Diagnosis of MetSy was based on NCEP ATP III—National Cholesterol Education Programs Adult Treatment Panel III criteria [14]. Patients diagnosed with MetSy formed the MetSy group (*n* = 65), while participants not fulfilling MetSy criteria comprised the control group (*n* = 65).

### 2.2. Risk Factor Assessment

Data on modifiable and non-modifiable cardiovascular (CV) risk factors for individual patients (age, gender, hypertension, hyperlipidemia, smoking, and diabetes) were retrieved from patients and their medical records. Diagnosis of hypertension was based on the patients’ medical history, blood pressure measurement (systolic blood pressure > 140 mmHg, and/or diastolic blood pressure > 90 mmHg), or history of antihypertensive therapy. Total cholesterol values > 5.0 mmol/L and/or triglycerides > 1.7 mmol/L were considered hyperlipidemic. The diagnosis of diabetes mellitus was based on patients’ medical history, fasting hyperglycemia, or the use of antidiabetic therapy (oral hypoglycemic drugs and/or insulin). Each patient’s risk factors were imputed into online calculators, SCORE and SCORE2 risk assessment models of the ESC Guidelines on cardiovascular disease prevention in clinical practice [15]. These risk assessment tools provide the estimated individual CV risk, including CV risk for specific geographic region, with Serbia indicated as a country with very high CV risk.

### 2.3. Anthropometric Measurements

Anthropometric measurements, including body weight and height, WC, and hip circumference, were obtained from all subjects while wearing light clothing without any footwear. These parameters provided an insight into nutrition status and the presence of abdominal obesity. BMI was calculated as weight (expressed in kilograms) divided by square of height (expressed in square meters), using the National Heart, Lung and Blood Institute (NHLBI) calculator [16]. Patients with BMI 25–29.9 kg/m^2^ were categorized as overweight, and BMI ≥ 30 kg/m^2^ as obese. Waist-to-hip-ratio was calculated by dividing the WC with the hips circumference. Total body surface area was determined according to the formula by Du Bois [17].

### 2.4. Laboratory Analyses

After the physical examination, 5 mL of blood was drawn from the cubital vein for standard laboratory analyses (total white blood cell count, glycemia, urea, creatinine, C reactive protein, alanine aminotransferase—ALT, aspartate aminotransferase—AST, gamma-glutamyl transferase—GGT, uric acid, albumin, total cholesterol, triglycerides, lipoprotein fractions—HDL and LDL cholesterol, and HbA1c). Analyses were performed using the standard laboratory techniques (spectrophotometry; latex turbidimetric essay (HgA1C); Biosystems BA 400). Ox-LDL-Chol, iNOS, and PAI-1 were measured using the ELISA (enzyme-linked immunosorbent assay) technique according to manufacturer’s instructions (Human iNOS ELISA Kit, Novusbio, Centennial, CO, USA; No NBP2-80255; Human Serpin E1/PAI-1 ELISA Kit, Novusbio, USA; No NBP2-60644; Human Oxidized LDL ELISA Kit, Novusbio, USA; No NBP2-79693). Levels of nitrates/nitrites (NO_2_^−^/NO_3_^−^) in plasma samples were determined using the Griess method [18]. Microalbuminuria was measured semiquantitatively using the Cybow2AC test strips.

### 2.5. Non-Invasive Assessment of Liver Disease

The presence of liver disease was evaluated using a simple algorithm for the prediction of hepatic steatosis—fatty liver index (FLI) [19]. FLI is calculated using the parameters routinely measured in clinical practice: BMI, WC, levels of triglycerides, and GGT. The values of FLI < 30 exclude fatty liver, while the FLI values ≥ 60 suggest possible fatty liver.

Severity of liver fibrosis was estimated using nonalcoholic fatty liver disease (NAFLD) fibrosis score, which combines 6 variables into a mathematical model discriminating subjects with advanced fibrosis (F3–F4) from those without advanced liver fibrosis (F1–F2) [20]. This scoring system uses the following variables: age; fasting glycemia, i.e., the presence of diabetes; AST/ALT ratio; BMI; platelets count; and albumin level.

### 2.6. Imaging Modalities

Color Doppler imaging of the carotid arteries was performed in all included subjects using the Acuson Sequoia C256 machine (Siemens Medical Solutions, Malvern, PA, USA), with a multi-frequency 4–13 MHz linear probe. Intraluminal lesions were assessed using the B-mode imaging and defined as intima-media complex (IMC) thickenings and plaques with focal intimal thickenings. The thickness of the IMC was measured in the posterior wall of the common carotid artery 2 cm distal from the bifurcation, in the region free from any focal changes. IMC thickness > 0.9 mm was considered pathological. Presence of atherosclerotic plaque was defined based on the Mannheim intima-media thickness consensus as an intraluminal prominence exceeding 1.5 mm, or a focal wall thickness increase of 0.5 mm, or a 50% increase in relation to the thickness of the adjacent IMC [21]. Longitudinal images of the common carotid artery and its branches, internal and external carotid artery, were analyzed bilaterally with the measurements of stenosis diameter and analysis of plaque characteristics. The interpretation was based on the combination of B-mode, i.e., real-time tissue imaging, and Doppler spectral analysis.

Additionally, standard 2D transthoracic echocardiography was performed in all patients.

This study complies with the Declaration of Helsinki and was performed according to approval of the Ethics Committee of Faculty of Medicine, University of Niš (approval no. 12-3182-2/6).

### 2.7. Statistical Analysis

Normal distribution of the data was tested quantitatively using the Shapiro–Wilk test, and graphically using histogram and Q-Q plot. Data with normal distribution were presented as mean and standard deviation, while non-normally distributed data were presented as median with interquartile range. Between -group difference was calculated using the chi-squared test, as well as Fisher’s test for categorical variables. Difference between the continuous variables with normal distribution was tested using Student’s *t*-test for independent samples and ANOVA, or with the Mann–Whitney U test and Kruskal–Wallis test for variables with non-normal distribution. Binary logistic regression analysis was performed to determine the predictors of NAFLD and asymptomatic atherosclerotic plaque in carotid arteries. All tests were performed as two-tailed, and *p*-value of <0.05 was considered statistically significant. Data analysis was performed in the statistical software package SPSS, version 25 (IBM Corporation, Armonk, NY, USA).

## 3. Results

The study involved 130 subjects, average age 52.2 ± 8.2 years, with 54% being male. Demographic and clinical characteristics of the study population are presented in Table 1. Subjects with MetSy had a higher average number of CV risk factors (*p* < 0.0001); higher CV risk score (both SCORE and SCORE2; *p* < 0.0001); and larger WC, BMI, and body surface area (*p* < 0.0001). Additionally, diabetes, hypertension, hyperlipidemia, and obesity were more prevalent among participants with MetSy (*p* < 0.001). Using the new model for the risk assessment of fatal and non-fatal cardiovascular disease—SCORE2, 74% of patients with MetSy were categorized as having a very high CV risk, and 25% of patients as having a high CV risk. Using the previous SCORE charts, a very high risk was found in 8% and high risk in 32% of the study population. All the obesity assessment parameters (BMI, WC, waist-to-hip ratio, and body surface area—BSA) were significantly higher in subjects with MetSy compared to the control group (*p* < 0.001 in all cases).

Based on the presence of obesity, patients with MetSy were divided into two subgroups (Group I, obese with BMI ≥ 30 kg/m^2^, and Group II, non-obese with BMI < 30 kg/m^2^), allowing their comparison, along with the comparison with the control group (Table 2). About 50% of subjects with MetSy were obese, showing no gender difference. Obese individuals from Group I had more MetSy components compared to non-obese individuals with MetSy. Obese patients with MetSy had significantly higher BMI, BSA, and waist-to-hip ratio compared to non-obese subjects (*p* < 0.01, *p* < 0.01, and *p* < 0.05, respectively). Participants with MetSy had higher values of obesity-related parameters (BMI, BSA, waist-to-hip ratio) compared to the control group, regardless of the obesity status.

Obese individuals with MetSy had significantly higher glycemia (*p* < 0.01), HbA1c (*p* < 0.05), and creatinine clearance (*p* < 0.05) compared to the non-obese MetSy group. Levels of triglycerides, remnant cholesterol, and uric acid were numerically higher in obese than in non-obese subjects with MetSy. Both groups with MetSy had higher values of glycemia, HbA1c, and all parameters of lipid status than control subjects (Table 3).

Levels of ox-LDL-Chol were significantly higher in patients with MetSy compared to control subjects. Levels of NO metabolites were similar in obese and non-obese individuals with MetSy (16.34 ± 11.73 vs. 14.12 ± 10.88, *p* > 0.05), while the control group had higher NO metabolite levels than both MetSy subgroups (33.85 ± 30.09, *p* < 0.001 for both comparisons). Inducible NOS and PAI-1 were significantly higher in patients MetSy compared to the control group, regardless of the obesity status, with iNOS being significantly higher in obese patients with MetSy compared to non-obese subjects with MetSy (115.93 ± 33.19 vs. 130.10 ± 35.84, *p* < 0.01). Levels of PAI-1 were numerically lower in obese subjects with MetSy than in the non-obese group (171.21 ± 48.85 vs. 149.21 ± 74.67, *p* > 0.05).

NAFLD was more prevalent in obese patients with MetSy, accompanied by higher FLI compared to non-obese subjects with MetSy (100% vs. 70%, *p* < 0.001, and 91.62 ± 8.98 vs. 65.30 ± 24.57, *p* < 0.001, respectively) (Table 4). Liver fibrosis assessed by NAFLD score was confirmed in four (12.5%) obese patients with MetSy, with no cases among non-obese subjects with MetSy nor in the control group.

Both groups of patients with MetSy had more prevalent asymptomatic carotid atherosclerosis than the control group, followed by higher values of IMC thickness, higher percentage of carotid artery stenosis, and numerous atherosclerotic plaques. LVMI had numerically highest values in obese patients with MetSy, while diastolic function was more prevalent in both MetSy groups than in controls (Table 4).

Hypertension, higher values of waist-to-hip ratio, and higher levels of PAI-1 and remnant cholesterol were significantly associated with the occurrence of NAFLD (Table 5).

Age, hypertension, and higher waist-to-hip ratio were significantly associated with the presence of asymptomatic atherosclerotic plaques in carotid arteries (Table 6).

## 4. Discussion

The present study comprehensively evaluated cardiovascular risk factors, anthropometric characteristics, numerous and diverse biochemical analyses, Doppler imaging characteristics, and the presence of NAFLD in a cohort of patients with and without MetSy. The main anthropometric parameters (BMI, BSA, and waist-to hip ratio) and glucoregulation indicators were similar in patients with MetSy, regardless of their obesity status, but higher than in patients without MetSy. On the other hand, obese and non-obese patients with MetSy had similar lipid profile, levels of ox-LDL Chol, NO metabolites, and PAI-1, with iNOS being significantly lower in obese subjects with MetSy than in the non-obese group.

Although easily calculated, BMI is considered as an insufficient measure of body fat content, since it does not involve muscle mass and bone density, nor does it reflect fatty tissue distribution. WC is usually used to identify abdominal obesity, while waist-to-hip ratio represents an effective measure of central adiposity, with both being associated with multiple harmful cardiometabolic outcomes [22,23] and both being significant predictors of CV events [24,25]. However, distribution of adipose tissue affects the health risk, and therefore the waist-to-hip ratio proved to be a more useful parameter for predicting MetSy in different populations [26,27,28]. Similar results were observed in our cohort of patients MetSy, especially the obese subgroup, which had the highest values of waist-to-hip ratio, along with BMI, WC, and body surface area.

Additionally, our study evaluated the association between obesity and NAFLD, asymptomatic atherosclerosis of the carotid arteries, and heart damage. Non-invasive estimation of NAFLD prevalence was possible due to the use of FLI, a screening tool often used to indicate the population at high risk of hepatic steatosis [19]. Using FLI in our study population, NAFLD was present in the majority of patients with MetSy (54 of 65, 83.1%), being more prevalent in the obese than in the non-obese MetSy group (100% vs. 70%, respectively). Large-scale meta-analysis of almost 5.4 individuals showed the estimated global NAFLD prevalence of 29.84%. The same study provided evidence of 0.7% global yearly increase in NAFLD prevalence, with the increase in 1.1% per year in Europe [29]. However, the prevalence of NAFLD was larger in patients with diabetes (58.9%) and obese participants (58.2%), which is concordant with our results. Taking into account the frequent co-existence of NAFLD and MetSy, using a simple method for estimating NAFLD in patients with MetSy [30] might be helpful in screening this broad population, as well as providing additional diagnostic methods for suspected cases.

Two prospective studies, Humedica HER [31] and The Health Improvement Network (THIN) [32] database, with the follow-up periods of 1.54 and 4.96 years, respectively, showed a positive linear relationship between the increase in BMI with the potential risk for NAFLD/NASH (nonalcoholic steatohepatitis) diagnosis. Consequently, the risk for NAFLD/NASH diagnosis was approximately five to nine times greater in patients with BMI 30–32.5 kg/m^2^, and about 10–14 times greater with BMI 37.5–40 kg/m^2^, compared with BMI ranging from 20 to 22.5 kg/m^2^ [33]. The relationship between NAFLD and BMI was again proven by a recently published study of Yari et al. [34], which included patients primarily with NAFLD and explored their cardiometabolic profile regarding the presence of obesity. It was shown that the prevalence of MetSy gradually increased in normal, overweight, and obese NAFLD patients (10.2%, 27.7%, and 62.1%, respectively), which is consistent with our results.

A panel of international experts have suggested a new term for NAFLD and introduced the acronym MAFLD—metabolic dysfunction-associated fatty liver disease. The goal behind this terminology change was to point to the close association between fatty liver; metabolic dysfunctions; and susceptibility to the development of diabetes, atherosclerosis, chronic kidney disease etc. [35]. The new definition was the result of a better insight and understanding of pathological processes leading to systemic metabolic dysfunction present in MAFLD, rather than excluding causes not leading to the disease (“nonalcoholic”). Current concept defines MAFLD as the presence of hepatic steatosis (confirmed by biopsy, imaging of blood biomarkers), accompanied by at least one of the following: overweight/obesity, type 2 diabetes, and proven metabolic dysregulation in normal weight subjects [35].

Our results demonstrated a high prevalence of MAFLD in both non-obese (70%) and obese (100%) patients with MetSy. Clinical significance of these findings indicates the excess body weight as a critical determinant of unfavorable clinical outcome in patients with MAFLD. Meta-analysis of 239 prospective studies has demonstrated the association between overweight and obesity with a higher risk of overall mortality, compared to patients with normal body weight [36]. Since MAFLD is usually encountered in overweight or obese individuals, accompanied by the increasing prevalence of overweight and obesity worldwide, screening of MAFLD would be useful in routine medical practice. Similarly, a close association between MAFLD and T2DM has also been shown, with >70% of patients with T2DM having MAFLD, implying required NAFLD screening in patients with diabetes [37].

In addition to routine lipid status, our study also analyzed remnant cholesterol in the study population. This parameter describes cholesterol content in triglyceride-rich lipoproteins, including non-LDL-C and high-density lipoprotein cholesterol (HDL-C) [38]. Numerous studies have reported the positive association between remnant cholesterol and the risk of coronary artery disease [39], NAFLD [40], type 2 diabetes [41], and metabolic syndrome [42]. After a median follow-up of 307 months, a study of Huang et al. [40] (2022) suggested that remnant cholesterol was independently associated with risks for MAFLD and death from all causes, CVD, and death caused by cancer in patients with MAFLD. These findings present a rationale for remnant cholesterol screening in individuals with high MAFLD risk and in those with diagnosed MAFLD. In our study, there was a significant association between the remnant cholesterol and NAFLD, which corroborated the results of the study reporting an independent association between remnant cholesterol and risk for NAFLD in the general population [43].

It has been known that the levels of PAI-1 are increased in patients with obesity and MetSy [13]. However, the data are scarce on the levels of PAI-1 in non-obese patients with MetSy. Our study showed non-significantly lower levels of PAI-1 in non-obese vs. obese subjects with MetSy (mean 149.1 vs. 171.2 ng/mL). Additionally, we have observed significant correlation between PAI-1 and NAFLD. This finding was confirmed by the results of meta-analysis, which included over 10,000 subjects, with the mean difference in PAI-1 levels of 17.147 between NAFLD and control subjects [44].

Patients with MetSy (obese and non-obese) had lower levels of NO metabolites compared to the control group, while previous studies showed conflicting results in assessing the relationship between nitric oxide products with MetSy [12,45]. On the other hand, levels of stress-induced NO-producing enzyme, iNOS, were significantly higher in patients with MetSy compared to the control group. During the pathogenesis of MetSy, there is a reduced endothelial NOS activity and NO bioavailability, consequently leading to endothelial dysfunction, with compensatory activation of iNOS, compensating the lack of NO, which is in line with our results [46].

## 5. Conclusions

The results of our study suggest that patients with MetSy have reduced levels of NO metabolites and increased levels of iNOS, ox-LDL-Chol, and PAI-1 compared to subjects without this metabolic disorder. Inducible NOS was significantly higher in obese patients with MetSy compared to the non-obese subgroup. The occurrence of NAFLD was significantly associated with the presence of hypertension, higher values of waist-to-hip-ratio, higher PAI-1, and remnant cholesterol levels. Given the high prevalence of NAFLD in patients with MetSy, NAFLD screening might be helpful in routine clinical practice and improve cardiometabolic and overall health of an enormous population of patients.

## Figures and Tables

**Table 1:** Demographic and clinical characteristics of the study population.

Characteristic	Total	MetSy Group (*n* = 65)	Control Group (*n* = 65)	*p*-Value
Age (years)	52.2 ± 8.2	53.9 ± 8.4	50.4 ± 7.7	0.015
Male gender (*n*, %)	71 (54.6%)	39 (60.0%)	33 (49.2%)	0.218
MetSy components, *n* (%)				
● 0	33 (25.4%)	0	33 (25.4%)	
● 1	10 (7.7%)	0	10 (7.7%)	
● 2	22 (16.9%)	0	22 (16.9%)	
● 3	21 (16.2%)	21 (16.2%)	0	
● 4	24 (18.5%)	24 (18.5%)	0	
● 5	20 (15.4%)	20 (15.4%)	0	
Hypertension, *n* (%)	55 (42.3%)	49 (76.0%)	6 (9.2%)	<0.001
Hyperlipidemia, *n* (%)	96 (74.0%)	65 (98.0%)	32 (49.0%)	<0.001
Diabetes mellitus, *n* (%)	20 (15.4%)	19 (29.0%)	1 (1.5%)	<0.001
Smoking, *n* (%)	33 (25.4%)	19 (29.2%)	14 (21.5%)	0.314
Risk factors, *n* (%)	2.1 ± 1.3	3.0 ± 0.9	1.1 ± 1.0	<0.001
Obesity, *n* (%)	44 (34.0%)	34 (52.0%)	11 (16.9%)	<0.001
Body weight (kg), *n* (%)	86.2 ± 20.5	94.4 ± 16.5	78.0 ± 20.8	<0.001
WC (cm), *n* (%)	98.9 ± 18.6	108.7 ± 13.5	89.1 ± 17.9	<0.001
HC (cm), *n* (%)	107.5 ± 12.1	112.7 ± 10.9	102.2 ± 10.9	<0.001
Waist-to-hip ratio, (mean ± SD)	0.91 ± 0.11	0.96 ± 0.09	0.87 ± 0.11	<0.001
BMI (kg/m^2^), (mean ± SD)	28.41 ± 5.56	31.06 ± 4.52	25.76 ± 10.94	<0.001
BSA, (mean ± SD)	1.99 ± 0.29	2.10 ± 0.22	1.89 ± 0.32	<0.001
SCORE, (mean ± SD)	3.88 ± 3.11	3.88 ± 3.11	1.45 ± 1.28	<0.001
SCORE 2, (mean ± SD)	9.98 ± 5.34	12.53 ± 4.94	7.44 ± 4.47	<0.001

SD—standard deviation; WC—waist circumference; HC—hip circumference; BSA—body surface area.

**Table 2:** Characteristics of patients with MetSy (obese and non-obese) and the control group.

Characteristics	I Group MetSy BMI ≥ 30 kg/m^2^ (*N* = 32)	II Group MetSy BMI < 30 kg/m^2^ (*N* = 33)	III Group, Control (*N* = 65)	*p*-Value
Age (years), (mean ± SD)	54.5 ± 8.7	53.4 ± 8.0	50.4 ± 7.7	I:II n.s. I:III *p* < 0.01 II:III *p* < 0.01
Male gender, *n* (%)	19 (59%)	20 (69%)	33 (49.2%)	I:II n.s. I:III n.s. II:III n.s
BMI (kg/m^2^), (mean ± SD)	34.44 ± 3.98	27.78 ± 1.72	25.76 ± 10.94	I:II *p* <0.001 I:III *p* < 0.001 II:III *p* < 0.001
BSA (m^2^), (mean ± SD)	2.19 ± 0.18	1.99 ± 0.22	1.89 ± 0.32	I:II *p* <0.001 I:III *p* < 0.001 II:III *p* < 0.01
Waist-to-hip ratio, (mean ± SD)	0.99 ± 0.10	0.94 ± 0.08	0.87 ± 0.11	I:II *p* < 0.05 I:III *p* < 0.001 II:III *p* < 0.001

MetSy—metabolic syndrome; BMI—body mass index; BSA—body surface area; n.s.—non-significant (*p* < 0.05).

**Table 3:** Biochemical analyses in patients with MetSy (obese and non-obese) and the control group.

Parameter	I Group MetSy BMI ≥ 30 kg/m^2^ (*N* = 32)	II Group MetSy BMI < 30 kg/m^2^ (*N* = 33)	III Group, Control (*N* = 65)	*p*-Value
Glycemia (mmol/L)	7.24 ± 1.80	6.04 ± 0.90	5.56 ± 0.55	I:II *p* < 0.01 I:III *p* < 0.001 II:III *p* < 0.01
HbA1c (%)	6.53 ± 1.35	5.84 ± 1.38	4.72 ± 1.04	I:II *p* < 0.05 I:III *p* < 0.001 II:III *p* < 0.001
Total cholesterol (mmol/L)	6.87 ± 1.59	6.83 ± 1.18	6.16 ± 1.03	I:II n.s. I:III *p* < 0.05 II:III *p* < 0.05
LDL cholesterol (mmol/L)	4.55 ± 1.07	4.53 ± 1.14	4.04 ± 0.98	I:II n.s. I:III *p* < 0.05 II:III *p* < 0.05
HDL cholesterol (mmnol/L)	1.13 ± 0.29	1.17 ± 0.24	1.57 ± 0.37	I:II n.s. I:III *p* < 0.001 II:III *p* < 0.001
Non-HDL cholesterol (mmnol/L)	5.74 ± 1.50	5.66 ± 1.12	4.51 ± 1.08	I:II n.s. I:III *p* < 0.001 II:III *p* < 0.001
Remnant cholesterol (mmnol/L)	1.29 ± 0.99	1.14 ± 0.38	0.55 ± 0.31	I:II n.s. I:III *p* < 0.001 II:III *p* < 0.001
Triglycerides (mmnol/L)	3.79 ± 2.84	2.75 ± 1.20	1.24 ± 0.66	I:II n.s. I:III *p* < 0.001 II:III *p* < 0.001
Uric acid (mmol/L)	385.07 ± 95.42	356.43 ± 74.76	269.19 ± 93.90	I:II n.s. I:III *p* < 0.001 II:III *p* < 0.001
Creatinine (µmol/L)	89.30 ± 15.13	93.08 ± 16.15	82.15 ± 12.35	I:II n.s. I:III *p* < 0.01 II:III *p* < 0.001
Creatinine clearance (mL/min)	109.53 ± 26.25	91.82 ± 22.06	95.87 ± 28.76	I:II *p* < 0.05 I:III *p* < 0.001 II:III n.s.
NO metabolites (µmol/L)	16.34 ± 11.73	14.12 ± 10.88	33.85 ± 30.09	I:II n.s. I:III *p* < 0.001 II:III *p* < 0.001
iNOS (pg/mL)	115.93 ± 33.19	130.10 ± 35.84	77.78 ± 50.10	I:II *p* < 0.01 I:III *p* < 0.001 II:III *p* < 0.05
Ox-LDL-Chol	1263.08 ± 326.57	1272.62 ± 430.60	954.37 ± 336.50	I:II n.s. I:III *p* < 0.001 II:III *p* < 0.01
PAI-1 (ng/mL)	171.21 ± 48.85	149.21 ± 74.67	88.25 ± 58.90	I:II n.s. I:III *p* < 0.001 II:III *p* < 0.01
CRP (mg/L)	3.39 ± 6.72	2.02 ± 4.94	1.63 ± 2.63	I:II n.s. I:III n.s. II:III n.s.

HbA1c—glycated hemoglobin; NO—nitric oxide; iNOS—inducible nitric oxide synthase; Ox-LDL-Chol—oxidized LDL cholesterol; PAI-1—plasminogen activator inhibitor-1; CRP—C-reactive protein; n.s.—non-significant (*p* > 0.05). All values are presented as mean ± standard deviation.

**Table 4:** Parameters of target organ damage in patients with MetSy (obese and non-obese) and in the control group.

Parameter	I Group MetSy BMI ≥ 30 kg/m^2^ (*N* = 32)	II Group MetSy BMI < 30 kg/m^2^ (*N* = 33)	III Group, Control (*N* = 65)	*p*-Value
NAFLD, *n* (%)	32 (100%)	23 (69.7%)	12 (18.5%)	I:II *p* < 0.001 I:III *p* < 0.001 II:III *p* < 0.001
FLI, Me (IQR)	95.0 (87.0–98.0)	76.5 (49.7–84.0)	23.0 (6.0–54.0)	I:II *p* < 0.001 I:III *p* < 0.001 II:III *p* < 0.001
IMC thickness (mm), (mean ± SD)	0.82 ± 0.22	0.77 ± 0.19	0.64 ± 0.12	I:II n.s. I:III *p* < 0.001 II:III *p* < 0.001
Presence of carotid plaques, *n* (%)	17 (53%)	22 (66.7%)	13 (20%)	I:II n.s. I:III *p* < 0.001 II:III *p* < 0.001
Number of carotid plaques, Me (IQR)	1.0 (0.0–2.0)	1.0 (0.0–2.0)	0.0 (0.0–0.0)	I:II n.s. I:III *p* < 0.001 II:III *p* < 0.001
Carotid stenosis, %, Me (IQR)	30.0 (0.0–35.0)	29.0 (0.0–40.0)	0.0 (0.0–0.0)	I:II n.s. I:III *p* < 0.001 II:III *p* < 0.001
Bilateral carotid plaques, *n* (%)	13 (40%)	10 (30%)	5 (7.7%)	I:II n.s. I:III *p* < 0.001 II:III *p* < 0.01
LVMI (g/m^2^), Me (IQR)	91.3 (79.6–108.0)	77.5 (71.2–100.0)	84.7 (68.9–97.2)	I:II n.s. I:III *p* < 0.01 II:III n.s.
Diastolic dysfunction, *n* (%)	15 (46.8%)	14 (42%)	10 (15%)	I:II n.s. I:III *p* < 0.001 II:III *p* < 0.01
E/a < 0.8, *n* (%)	11 (34.4%)	10 (30.3%)	6 (9.2%)	I:II n.s. I:III *p* < 0.01 II:III *p* < 0.01
E/a 0.8–2.0, *n* (%)	21 (65.6%)	23 (69.7%)	59 (90.8%)	
DP (mmHg/min), Me (IQR)	10,920.0 (9750.0–11,960.0)	10,070.0 (8927.5–12,340.0)	10,300.0 (9367.5–12,180.0)	I:II n.s. I:III n.s. II:III n.s.

FLI—fatty liver index; IMC—intima media complex; LVMI—left ventricular mass index; DP—double product (heart rate × systolic blood pressure); Me—median, (IQR)—interquartile range; n.s.—non-significant (*p* < 0.05).

**Table 5:** Factors associated with NAFLD in overall study population.

Characteristics	OR (95% CI)	*p*-Value
Age (years)	1.111 (0.984–1.254)	0.090
Male gender	0.587 (0.052–6.675)	0.668
Hypertension	7.943 (1.032–61.130)	0.047
Waist-to hip ratio 0.88–0.97 *	24.997 (1.963–318.172)	0.013
Waist-to-hip ratio 0.98–1.16 *	73.117 (4.250–1259.936)	0.003
NO	0.954 (0.897–1.015)	0.136
iNOS	0.992 (0.974–1.011)	0.399
PAI-1	1.025 (1.006–1.043)	0.008
Remnant cholesterol	102.436 (6.565–1598.297)	0.001

* Reference group: waist-to-hip ratio 0.68–0.87; NO—nitric oxide; iNOS—inducible nitric oxide synthase; Hosmer–Lemeshow test *p* = 0.856.

**Table 6:** Factors associated with the presence of asymptomatic atherosclerotic plaques in carotid arteries in the overall study population.

Characteristics	OR (95% CI)	*p* Value
Age (years)	1.123 (1.053–1.197)	<0.001
Male gender	1.460 (0.422–5.048)	0.550
Hypertension	7.127 (1.865–27.238)	0.004
Waist-to-hip ratio 0.88–0.97 *	5.134 (1.126–23.420)	0.035
Waist-to-hip ratio 0.98–1.16 *	4.238 (1.702–10.554)	0.002
NO	0.611 (0.167–2.243)	0.458
iNOS	0.561 (0.193–1.634)	0.290
PAI-1	1.123 (1.053–1.197)	<0.001
Remnant cholesterol	1.460 (0.422–5.048)	0.550

* Reference group: waist-to-hip ratio 0.68–0.87; NO—nitric oxide; iNOS—inducible nitric oxide synthase; Hosmer–Lemeshow test *p* = 0.297.

## Data Availability

Research data are not publicly available due to privacy restrictions.
